# Intraperitoneal Fetuin-A Modulates HMGB1-Associated Inflammatory Remodeling in Experimental Endometriosis

**DOI:** 10.3390/biomedicines14102149

**Published:** 2026-09-23

**Authors:** Selim Afşar, Özgür Bulmuş, Figen Efe Çamili, Mustafa Hilmi Yaranoğlu, Mine İslimye Taşkın, Ayla Solmaz Avcıkurt, Gürhan Güney, Ezgi Tolu Cenk, Hilmi Bolat, Ceyda Sancaklı Usta, Merve Akış Yılmaz, Akın Usta, Gülay Turan

**Affiliations:** 1 Department of Obstetrics & Gynecology, Faculty of Medicine, Balıkesir University, 10020 Balıkesir, Turkey; figen.camili@balikesir.edu.tr (F.E.Ç.); gurhanguney@yahoo.com (G.G.);; 2 Department of Physiology, Faculty of Medicine, Balıkesir University, 10020 Balıkesir, Turkey; ozgur.bulmus@balikesir.edu.tr; 3 Experimental Animals Production, Care, Application and Research Center, Balıkesir University, 10020 Balıkesir, Turkey; mhyaranoglu@balikesir.edu.tr; 4 Department of Medical Biology, Faculty of Medicine, Balıkesir University, 10020 Balıkesir, Turkey; aylaavcikurt@hotmail.com (A.S.A.); hilmi.bolat@balikesir.edu.tr (H.B.); 5 Department of Obstetrics & Gynecology, Balıkesir City Hospital, 10100 Balıkesir, Turkey; ezgitolu@gmail.com; 6 Department of Biochemistry, Faculty of Medicine, Balıkesir University, 10020 Balıkesir, Turkey; merve.akis@balikesir.edu.tr; 7 Department of Pathology, Faculty of Medicine, Balıkesir University, 10020 Balıkesir, Turkey; gulaytr@yahoo.com.tr

**Keywords:** endometriosis, Fetuin-A, HMGB1, inflammation, tissue remodeling, autophagy, rat model

## Abstract

**Background/Objectives:** Endometriosis is a chronic inflammatory disorder characterized by ectopic endometrial-like lesions, inflammatory activation, angiogenesis, and tissue remodeling. Fetuin-A has been implicated in inflammatory regulation; however, its effects on *HMGB1* mRNA expression and lesion-remodeling markers in endometriosis remain unclear. This study investigated whether intraperitoneal Fetuin-A is associated with changes in systemic inflammation and lesion-associated remodeling in a surgically induced rat model of endometriosis. **Methods:** After confirmation of viable endometriotic implants, female Wistar albino rats received intraperitoneal Fetuin-A or saline. Serum IL-1β, IL-6, TNF-α, and AMH levels were measured. Endometriotic implants were evaluated by qRT-PCR for *HMGB1* and *OPN* mRNA expression and by immunohistochemistry for Fetuin-A, p62/SQSTM1, caspase-3, VEGF, OPN, and SOD1. **Results:** For the primary lesion-area endpoint, Fetuin-A treatment remained associated with significantly lower post-treatment lesion area after adjustment for baseline lesion area. Compared with saline, Fetuin-A treatment significantly reduced serum IL-1β, IL-6, and TNF-α concentrations and was associated with lower AMH levels. In endometriotic implants, Fetuin-A was associated with marked downregulation of *HMGB1* mRNA expression, whereas *OPN* mRNA was modestly increased. Immunohistochemistry showed increased Fetuin-A, p62/SQSTM1, caspase-3, and SOD1 staining, together with reduced VEGF and OPN staining after Fetuin-A treatment. **Conclusions:** Intraperitoneal Fetuin-A attenuated experimental endometriosis, with a significant baseline-adjusted reduction in the primary lesion-area endpoint, systemic inflammatory suppression, and reduced *HMGB1* mRNA expression, accompanied by changes in angiogenesis-, oxidative stress-, apoptosis-, autophagy-, and remodeling-associated markers. Because HMGB1 protein/release, RAGE/TLR4/NF-κB signaling, and specific fibrosis markers were not assessed, these findings should not be interpreted as direct HMGB1 pathway inhibition or definitive anti-fibrotic activity. Overall, these results support further mechanistic studies evaluating Fetuin-A as a potential modulator of inflammatory and lesion-remodeling processes in endometriosis.

## 1. Introduction

Endometriosis is a chronic, estrogen-dependent inflammatory disorder characterized by the presence of endometrial-like tissue outside the uterine cavity. It affects up to 10% of reproductive-aged women worldwide and is frequently associated with dysmenorrhea, chronic pelvic pain, dyspareunia, infertility, fatigue, and reduced quality of life [1,2]. Although considered a benign disease, endometriotic lesions may display biological features that support persistence, including local invasion, angiogenesis, immune escape, oxidative stress, and recurrent tissue remodeling [3,4]. In addition, epidemiological evidence suggests a modest but reproducible association between endometriosis and selected ovarian cancer histotypes, particularly endometrioid and clear-cell ovarian carcinoma, emphasizing the relevance of chronic inflammation and remodeling-related mechanisms in disease progression [5].

Lesion persistence is strongly influenced by the inflammatory peritoneal microenvironment [4,6]. Recurrent inflammatory stimulation promotes cytokine release, vascular activation, cellular stress responses, and extracellular matrix remodeling, thereby favoring ectopic tissue survival. Fibrosis is increasingly recognized as a central pathological component of endometriosis, contributing to pain, adhesions, anatomical distortion, and reduced responsiveness to treatment [7,8]. However, the molecular pathways linking inflammation, fibrosis, and lesion persistence remain incompletely characterized.

Although endometriosis and polycystic ovary syndrome (PCOS) have traditionally been regarded as distinct reproductive disorders associated with different endocrine profiles, accumulating evidence indicates that they may share several pathogenic mechanisms. Both conditions have been linked to chronic low-grade inflammation, oxidative stress, immune dysregulation, ovarian microenvironmental dysfunction, fibrotic remodeling, and abnormalities in intracellular stress-response pathways, including autophagy [9,10]. These overlapping biological features support the concept that reproductive disorders may involve intersecting inflammatory and metabolic networks rather than entirely isolated disease processes; nevertheless, their hormonal milieu, clinical presentation, and disease-specific pathways remain clearly different.

High mobility group box 1 (HMGB1) is a damage-associated molecular mediator that links tissue injury, innate immune activation, and remodeling. Following extracellular release during cellular stress, HMGB1 activates Toll-like receptor- and receptor for advanced glycation end products (RAGE)-dependent signaling, amplifying pro-inflammatory cytokine production and downstream stromal responses [11]. Experimental and translational studies increasingly support HMGB1 as an important regulatory node within the inflammatory microenvironment of endometriosis [12,13]. However, HMGB1 should not be viewed as an isolated upstream regulator, because inflammation, fibrosis, angiogenesis, oxidative stress, apoptosis, and autophagy are regulated through multiple overlapping molecular pathways that dynamically interact during lesion establishment and progression.

Osteopontin (OPN) is a multifunctional matricellular glycoprotein involved in immune-cell recruitment, cell adhesion, extracellular matrix turnover, angiogenesis, and epithelial–mesenchymal transition. Elevated OPN expression has been reported in endometriotic tissues and may contribute to lesion implantation, persistence, and remodeling through interactions with inflammatory cytokines and extracellular matrix signaling pathways [13,14]. Importantly, HMGB1 and OPN may participate in overlapping inflammatory and tissue-remodeling networks, suggesting that simultaneous evaluation of both molecules may provide insight into how inflammatory suppression translates into changes in lesion-associated remodeling processes. Thus, OPN was included in the present study as a remodeling-associated marker rather than as a stand-alone fibrotic mediator.

Autophagy has emerged as an important but incompletely understood process in endometriosis pathobiology. Several studies suggest that autophagy may promote survival of ectopic endometrial cells exposed to hypoxic, inflammatory, and oxidative microenvironments by enhancing intracellular stress adaptation and metabolic recycling [15,16]. Conversely, other data indicate that autophagic activation may contribute to cellular turnover or lesion regression under specific conditions, emphasizing the context-dependent nature of autophagic responses [16]. Accordingly, autophagy in endometriosis is increasingly viewed as a dynamic regulatory process rather than a uniformly protective or pathogenic mechanism. HMGB1 is also a recognized regulator of autophagy signaling, positioning it within a broader network linking inflammation, tissue remodeling, cellular stress responses, and lesion persistence [17].

Fetuin-A is a liver-derived negative acute-phase glycoprotein with pleiotropic effects on inflammation, mineralization, fibrosis, tissue repair, and cellular stress responses [18,19,20,21]. Beyond systemic inflammatory disorders, emerging evidence suggests that Fetuin-A participates in reproductive and metabolic disease pathways characterized by chronic inflammation and altered tissue remodeling. Serum Fetuin-A alterations have been reported in infertility-related contexts, supporting its potential relevance to reproductive conditions characterized by oxidative stress and inflammatory imbalance [22]. Experimental studies have also shown that Fetuin-A may suppress HMGB1 release and attenuate HMGB1-dependent inflammatory signaling, thereby limiting cytokine amplification and tissue-injury responses [20]. Given the central contribution of inflammatory activation, fibrosis, angiogenesis, oxidative stress, and remodeling to endometriosis progression, Fetuin-A represents a biologically plausible candidate for modulating disease-associated molecular pathways. However, its effects on HMGB1-associated inflammatory and remodeling responses in endometriosis have not been adequately explored.

To our knowledge, experimental evidence regarding the effects of Fetuin-A on HMGB1-associated inflammatory and remodeling responses in endometriosis remains limited. The novelty of the present study lies not only in evaluating intraperitoneal Fetuin-A administration in a surgically induced endometriosis model but also in simultaneously examining systemic inflammatory cytokines, *HMGB1* mRNA expression, OPN-associated remodeling responses, angiogenesis-related markers, oxidative stress, apoptosis, and autophagy-associated signaling. This integrated approach was designed to provide a broader understanding of how Fetuin-A may influence interconnected biological pathways involved in lesion persistence.

Accordingly, this study evaluated the effects of intraperitoneal Fetuin-A administration on systemic inflammatory markers, HMGB1 expression, and remodeling-associated molecular, histopathological, and immunohistochemical findings in a surgically induced rat model of endometriosis. We hypothesized that Fetuin-A would attenuate HMGB1-associated inflammatory activity and modulate remodeling-associated responses, including angiogenesis-, oxidative stress-, apoptosis-, and autophagy-associated changes within endometriotic implants, while recognizing that these pathways represent interconnected biological networks rather than a single linear cascade.

## 2. Materials and Methods

### 2.1. Animals and Experimental Design

This experimental animal study was conducted at the Balıkesir University Experimental Animals Production, Care, Application and Research Center (BAUN-DEHAM). Twenty adult female Wistar albino rats, 3–4 months of age and weighing 250–300 g, were obtained from and housed at BAUN-DEHAM, an institutional laboratory animal production and care facility affiliated with Balıkesir University. All 20 animals completed model establishment, treatment allocation, and final analysis. No animals were excluded after model creation, and the final analyzed groups consisted of 10 rats in the Fetuin-A group and 10 rats in the saline group. No privately owned animals from another institution, individual, or farm were used. Animals were housed in standard laboratory cages under controlled environmental conditions, including room temperature of approximately 20 ± 2 °C, relative humidity of 50 ± 10%, and a 12-h light/dark cycle, with free access to standard chow and water. Before model induction, animals were allowed to acclimatize to the laboratory environment and were monitored for general health status.

The experimental workflow consisted of surgical induction of endometriosis, confirmation of viable endometriotic implants, random allocation to treatment or control groups, short-course intraperitoneal administration of Fetuin-A or saline, and subsequent collection of blood and tissue samples for biochemical, histopathological, immunohistochemical, and molecular analyses.

All procedures were approved by the Balıkesir University Local Animal Experiments Ethics Committee (Balıkesir, Türkiye; Approval No. 2021/4-12; Date: 29 April 2021) and were carried out in accordance with national regulations and international standards for laboratory animal care. Because the animals were institutionally bred and supplied by BAUN-DEHAM and were not privately owned by another institution, individual, or farm, owner informed consent was not applicable. Reporting followed the ARRIVE 2.0 guidelines [23].

### 2.2. Surgically Induced Endometriosis Model

Experimental endometriosis was induced using a surgical uterine autotransplantation model based on the method described by Vernon and Wilson, with minor protocol adaptations [24]. Rats were anesthetized with intraperitoneal ketamine (80–100 mg/kg; Ketasol 10%, VetViva Richter GmbH, Wels, Austria) and xylazine (8–10 mg/kg; Xylazinbio 2%, Interhas Tıbbi ve Kimyevi Ürünler San. ve Tic. A.Ş., Ankara, Türkiye). After abdominal skin preparation and sterile draping, a midline laparotomy was performed. One uterine horn was exteriorized and excised, rinsed with sterile saline, and opened longitudinally to expose the endometrial surface.

The uterine tissue was divided into approximately 0.5 × 0.5 cm fragments. Each fragment was sutured to the inner abdominal wall using 4/0 Vicryl (polyglactin 910; Ethicon, Inc., Somerville, NJ, USA), with the endometrial surface oriented toward the peritoneal cavity to facilitate ectopic implantation. The abdominal wall and skin were closed in layers, and animals were observed during recovery and postoperative follow-up.

Four weeks after the initial procedure, a second laparotomy was performed under anesthesia to verify lesion establishment. Endometriotic implants were inspected macroscopically for vascularization, cystic appearance, and attachment to the abdominal wall. All animals demonstrated viable, vascularized implants at second-look laparotomy and proceeded to treatment allocation; no animals were excluded after model creation.

### 2.3. Fetuin-A Administration

After confirmation of viable endometriotic implants, rats were randomly allocated to the Fetuin-A treatment group or saline control group using a computer-generated allocation sequence. The Fetuin-A group received intraperitoneal Fetuin-A (100 mg/kg; fetuin from fetal bovine serum, lyophilized powder; Sigma-Aldrich, St. Louis, MO, USA; Cat. No. F3004), whereas the control group received an equivalent intraperitoneal volume of saline (Serum Fizyolojik Biofarma 0.9%, 10 mL ampoules; Biofarma İlaç Sanayi ve Ticaret A.Ş., İstanbul, Türkiye). Injections were administered once daily for four consecutive days according to the experimental protocol.

Twenty-four hours after the final injection, animals were anesthetized and underwent terminal laparotomy. Blood samples were obtained by cardiac puncture for serum analysis, and endometriotic implants were excised for macroscopic inspection, histopathology, immunohistochemistry, and qRT-PCR analysis. Investigators performing histopathological and immunohistochemical scoring were blinded to group allocation.

### 2.4. Macroscopic and Histopathological Evaluation

After excision, endometriotic implants were inspected macroscopically, their dimensions were recorded with a calibrated ruler, and the specimens were then fixed in 10% neutral buffered formalin, processed through routine paraffin-embedding procedures, and cut into 5 μm sections. Hematoxylin and eosin-stained slides were reviewed to verify the presence of endometriotic tissue, defined by endometrial-type glands and stromal elements, and to assess lesion-associated remodeling features, including edema, vascularity, inflammatory cell infiltration, stromal organization, and fibrosis. Microscopic evaluation was performed with an Olympus BX48 light microscope (Olympus Corporation, Tokyo, Japan), and digital semi-quantitative assessment was supported by NIS-Elements Advanced Research Microscope Imaging Software (Nikon Instruments Europe BV, Amsterdam, The Netherlands; https://www.microscope.healthcare.nikon.com/en_EU/products/software/nis-elements/nis-elements-advanced-research, accessed on 21 September 2026).

Immunohistochemical staining was performed on 5 μm formalin-fixed, paraffin-embedded tissue sections. After deparaffinization and rehydration, heat-induced epitope retrieval was performed using citrate buffer or EDTA buffer according to antibody datasheet recommendations and local laboratory optimization. Endogenous peroxidase activity and nonspecific background staining were blocked before primary antibody incubation. The Santa Cruz Biotechnology antibodies (Santa Cruz Biotechnology, Inc., Dallas, TX, USA)—Fetuin-A (clone H-4, Cat. No sc-133146), p62/SQSTM1 (clone A-6, Cat. No. sc-48402), caspase-3 (clone 3C119, Cat. No. sc-70497), VEGF (Clone C-1, Cat. No. sc-7269), and OPN (clone LFMb-14, Cat. No. sc-73631)—were used within the manufacturer-recommended IHC-P dilution range of 1:50–1:500, with final working dilutions optimized according to tissue staining quality. The Abcam SOD1 antibody (ab13498) was used within the manufacturer-recommended IHC-P dilution range of 1:100–1:500, with final working dilution optimized according to tissue staining quality. Negative controls were prepared by omitting the primary antibody and processing sections through the same detection protocol. Positive-control tissues were included for each staining run as follows: rat liver tissue for Fetuin-A, rat liver for p62/SQSTM1, tonsil for caspase-3, placenta for VEGF, placenta for OPN, and liver for SOD1. Detection was performed using a polymer-based horseradish peroxidase/diaminobenzidine system, followed by hematoxylin counterstaining. Staining intensity and localization were evaluated semi-quantitatively by a blinded histopathologist.

Staining results were scored semi-quantitatively by considering both staining intensity and localization within glandular epithelial and stromal compartments. Immunoreactivity was graded as 0, absent; 1, weak; 2, moderate; 3, strong. To minimize assessment bias, all histopathological and immunohistochemical slides were evaluated by a single histopathologist who was unaware of the treatment groups.

### 2.5. RNA Isolation and Quantitative Real-Time PCR

Total RNA was extracted from paraffin-embedded endometriotic tissue using Wizol™ Reagent (WISBIOsolution, Seongnam-si, Republic of Korea) according to the manufacturer’s protocol. RNA concentration and purity were assessed spectrophotometrically before complementary DNA (cDNA) synthesis; all samples met acceptable NanoDrop purity criteria, with A260/A280 ratios ranging from 1.7 to 2.0 and A260/A230 ratios ranging from 2.0 to 2.2. Samples suitable for downstream analysis were reverse-transcribed using the OneScript^®^ Plus cDNA Synthesis Kit (Applied Biological Materials Inc., Richmond, BC, Canada, Cat. No. G236).

Quantitative real-time PCR was performed using BlasTaq™ 2× qPCR Master Mix (Applied Biological Materials Inc., Cat. Nos. G891 and G892) on a Rotor-Gene Q platform (Qiagen, Düsseldorf, Germany). Reactions were performed in triplicate. *HMGB1* and *OPN* expression levels were normalized to β-actin (*ACTB*), and relative expression was calculated using the 2^−ΔΔCt^ method with Rotor-Gene Q Software version 2.3.4 [25].

Primer sequences for *HMGB1*, *OPN*, and β-actin (*ACTB*) are shown in Table 1. All primers were used for gene-expression analysis after confirmation of sequence orientation and expected amplicon size, and β-actin served as the internal reference gene for normalization.

Primer efficiency analyses, formal reference-gene validation using additional housekeeping genes, no-template controls, no-reverse-transcription controls, and RNA integrity metrics were not available in the archived experimental records and therefore could not be retrospectively reported. Relative gene-expression results should therefore be interpreted within this methodological limitation.

### 2.6. Biochemical Analysis

Blood samples collected at sacrifice were centrifuged, and serum was separated and stored at −80 °C until analysis. Serum interleukin-1β (IL-1β), interleukin-6 (IL-6), and tumor necrosis factor-α (TNF-α) concentrations were measured to evaluate systemic inflammatory activity using commercially available rat ELISA kits: Rat IL-1 beta ELISA Kit (ab255730; Abcam, Cambridge, UK), Rat IL-6 ELISA Kit (ab234570; Abcam), and Rat TNF alpha ELISA Kit (ab46070; Abcam). Serum anti-Müllerian hormone (AMH) was measured as an endocrine marker related to ovarian reserve using the Rat AMH ELISA Kit (ab323518; Abcam). All assays were performed according to the manufacturer’s instructions.

### 2.7. Statistical Analysis

Statistical analyses were performed using Jamovi software (version 2.3.21). Continuous variables were summarized as mean ± SD. Data distribution was evaluated using the Shapiro–Wilk test, and variance homogeneity was considered when selecting the appropriate comparative test. Between-group comparisons were performed using Student’s *t*-test, Welch’s *t*-test, or Welch’s ANOVA, as appropriate for variance structure and comparison design. The baseline-adjusted lesion-area analysis was defined as the primary endpoint and was analyzed using ANCOVA, with the day-4 lesion area as the dependent variable, the treatment group as the fixed factor, and the baseline lesion area as a covariate. The baseline-by-treatment interaction was tested to evaluate the homogeneity-of-regression-slopes assumption. Unadjusted change-score comparisons were retained as supportive descriptive analyses. Multiplicity across the secondary biological endpoints, including serum cytokines, AMH, qRT-PCR markers, and immunohistochemical scores, was addressed using the Benjamini–Hochberg false-discovery-rate procedure, with Bonferroni correction considered as a conservative sensitivity approach; adjusted *p*-values are provided in Supplementary Table S1. No documented a priori sample-size calculation was available. The study analyzed 20 animals, with 10 animals per group, and this absence is acknowledged as a limitation. For the primary baseline-adjusted lesion-area ANCOVA, the estimated treatment effect was −37.57 area units (95% CI, −51.44 to −23.69; *p* < 0.001). Although the observed-effect retrospective power was 99.97%, this value was not used to justify study adequacy because observed power is determined by the observed effect estimate and *p*-value. In a prospective sensitivity analysis, an effect equal to 50% of the observed adjusted difference would require 11 analyzable animals per group to achieve 82.1% power. Thus, the study provides evidence for the large observed primary lesion-area effect but cannot be assumed to be adequately powered for smaller effects or for secondary outcomes. Within-group before/after comparisons were interpreted descriptively and were not considered sufficient to establish a treatment effect without the corresponding between-group adjusted analysis. A two-tailed *p*-value < 0.05 was considered statistically significant.

## 3. Results

### 3.1. Effect of Fetuin-A on Endometriotic Lesion

Baseline lesion area was numerically larger in the Fetuin-A group than in the saline group (92.1 vs. 67.4 area units; Welch’s *p* = 0.512). Therefore, the primary lesion-area endpoint was analyzed using ANCOVA, with the day-4 lesion area as the outcome, the treatment group as the fixed factor, and the baseline lesion area as the covariate. Fetuin-A treatment was associated with a significantly lower adjusted day-4 lesion area than saline treatment (adjusted mean difference −37.57 area units; 95% CI −51.44 to −23.69; *p* < 0.001; adjusted means: 27.12 vs. 64.68). The baseline-by-treatment interaction was not significant (*p* = 0.871), supporting the ANCOVA assumption. Unadjusted within-group changes were retained as supportive descriptive analyses only (saline: 67.4 to 59.4, *p* = 0.263; Fetuin-A: 92.1 to 32.4, *p* = 0.018) (Figure 1).

### 3.2. Effect of Fetuin-A on Systemic Inflammatory Markers and Ovarian Reserve

Fetuin-A administration was associated with a significant reduction in systemic inflammatory activity compared with saline-treated controls. Serum IL-1β, IL-6, and TNF-α concentrations were all lower in the Fetuin-A group, demonstrating a consistent anti-inflammatory response (all *p* ≤ 0.002). AMH levels were also significantly reduced in the Fetuin-A group (*p* < 0.001), and this endocrine marker was analyzed as a secondary biological endpoint alongside the inflammatory markers (Figure 2).

### 3.3. Effect of Fetuin-A on HMGB1 and OPN mRNA Expression

Welch’s ANOVA identified significant between-group differences in endometriotic implant gene expression. *HMGB1* mRNA expression was markedly lower in the Fetuin-A group than in the saline group (*p* < 0.001), indicating treatment-associated reduction in *HMGB1* transcript-level expression rather than direct evidence of HMGB1 pathway inhibition. In contrast, *OPN* mRNA expression was significantly higher in the Fetuin-A group than in saline-treated controls (*p* = 0.004), suggesting differential regulation of inflammatory and matrix-remodeling pathways at the transcript level (Figure 3).

### 3.4. Histopathological and Immunohistochemical Analysis

Histological examination confirmed endometriotic lesion formation in the experimental groups, with endometrial-type glands and stromal components identified on hematoxylin and eosin-stained sections. Semi-quantitative immunohistochemical scoring showed a distinct treatment-associated pattern. Compared with the saline group, Fetuin-A-treated implants had higher scores for Fetuin-A immunoreactivity (1.70 vs. 1.00), p62/SQSTM1 (2.00 vs. 1.00), caspase-3 (1.40 vs. 0.80), and SOD1 (2.20 vs. 1.20). Conversely, VEGF and OPN immunohistochemical staining scores were lower in the Fetuin-A group than in the saline group (VEGF: 1.10 vs. 2.20; OPN: 1.10 vs. 2.10), indicating reduced angiogenic and OPN protein-level staining responses (Figure 4).

Representative immunohistochemical sections supported the scoring results. Fetuin-A immunoreactivity was stronger in Fetuin-A-treated implants than in saline-treated controls (Supplementary Figure S1). OPN and VEGF staining were more prominent in saline-treated controls (Supplementary Figures S2 and S7). p62/SQSTM1 staining was stronger in Fetuin-A-treated implants than in saline-treated controls (Supplementary Figures S3 and S4), and caspase-3 and SOD1 staining were also increased in the Fetuin-A group (Supplementary Figures S5 and S6).

## 4. Discussion

The present study demonstrates that intraperitoneal Fetuin-A administration produces a coordinated anti-inflammatory and tissue-remodeling response in a surgically induced rat model of endometriosis. Fetuin-A treatment significantly reduced circulating IL-1β, IL-6, and TNF-α levels and was accompanied by marked downregulation of *HMGB1* mRNA expression in endometriotic implants. Together with reductions in VEGF and OPN immunohistochemical staining and increases in p62/SQSTM1, SOD1, caspase-3, and Fetuin-A immunoreactivity, these findings suggest that Fetuin-A may influence several interconnected processes involved in lesion persistence, including inflammatory activation, angiogenic signaling, oxidative stress regulation, autophagy-associated responses, apoptosis, and extracellular matrix remodeling.

Endometriosis is sustained by a chronic inflammatory microenvironment in which cytokines, damage-associated molecular mediators, immune cells, and stromal elements interact to support lesion survival [1,2,3,4,5,6]. Fibrosis is now regarded as an important pathological component of endometriosis, closely linked to adhesions, pain, anatomical distortion, and persistent tissue remodeling [7,8]. IL-1β, IL-6, and TNF-α contribute to inflammatory amplification, angiogenesis, nociceptive signaling, and resistance to apoptosis. The significant reduction in all three cytokines after Fetuin-A treatment indicates a systemic anti-inflammatory effect that is biologically relevant to experimental endometriosis and may secondarily reduce pro-remodeling inflammatory pressure within lesions. This effect is consistent with broader evidence that Fetuin-A can modulate inflammatory responses and tissue-injury pathways in experimental and systemic inflammatory settings [18,19,20,21].

A central finding of this study was the pronounced suppression of *HMGB1* mRNA expression in Fetuin-A-treated implants. HMGB1 is released during cellular stress and injury and can activate inflammatory pathways through pattern-recognition receptors, thereby linking tissue damage to cytokine production, immune-cell recruitment, autophagy-related signaling, and fibrotic remodeling [11,12,13,17]. In endometriosis, such signaling may contribute to lesion maintenance by reinforcing inflammatory and stromal activation, processes that are consistent with the recognized pro-fibrotic nature of the disease [7,8]. Because HMGB1 protein abundance, extracellular release, receptor activation, and downstream RAGE/TLR4/NF-κB signaling were not assessed, the present findings should be interpreted as evidence of altered *HMGB1* transcript-level expression rather than proof of direct inhibition of the HMGB1 signaling pathway.

The histopathological and immunohistochemical findings further support this interpretation. Evaluation of edema, vascularity, inflammatory infiltration, stromal organization, and fibrosis provided morphological context for the molecular results. Lower VEGF staining in Fetuin-A-treated implants suggests attenuation of angiogenic signaling, a key requirement for ectopic lesion survival and expansion. Increased caspase-3 immunoreactivity may reflect enhanced apoptotic activity, whereas higher SOD1 staining may indicate a compensatory antioxidant response or improved oxidative stress handling. The increase in p62/SQSTM1 immunoreactivity suggests altered autophagy-associated signaling, consistent with the known interaction between HMGB1 and autophagy-related pathways [17]; however, p62/SQSTM1 alone cannot establish autophagic flux, and this result should be interpreted as an indicator of pathway involvement rather than definitive evidence of increased or impaired autophagy.

An apparently divergent OPN pattern was observed: *OPN* mRNA expression increased in Fetuin-A-treated implants, whereas OPN immunohistochemical staining scores were lower. This discrepancy remains unresolved and should be interpreted cautiously. Because OPN protein levels were not quantified by Western blot or ELISA, and because paired individual-level correlation between *OPN* mRNA expression and immunohistochemical staining was not performed, the present data do not allow a causal explanation for the discordant transcript- and protein-level findings. Potential explanations include differences between whole-tissue transcript abundance and localized protein staining, post-transcriptional regulation, protein turnover, post-translational modification, tissue compartmentalization, antibody-based detection characteristics, or timing of sampling; however, these possibilities remain speculative. In addition, Ho et al. showed that OPN biology in endometriosis involves splice variants and contributes to proliferation, migration, and epithelial–mesenchymal transition in endometrial epithelial cells, supporting the view that OPN regulation is complex and cannot be fully captured by a single mRNA-versus-immunohistochemistry comparison [14]. Therefore, the OPN results require cautious interpretation and validation in future studies using quantitative protein assays and paired transcript–protein analyses.

Importantly, the present study did not directly quantify fibrosis using collagen-related markers such as *COL1A1*, *COL3A1*, α-SMA, TGF-β1, SMAD2/3, fibronectin, or fibrosis-specific stains such as Masson’s trichrome or Sirius Red. Therefore, reduced OPN or VEGF staining should be interpreted as evidence of treatment-associated changes in remodeling and angiogenic marker expression, not as definitive proof of reduced fibrosis.

The reduction in AMH observed in the Fetuin-A group requires cautious interpretation. Although AMH was analyzed as a secondary biological endpoint with multiplicity adjustment, the current study was not designed to determine whether Fetuin-A directly affects ovarian reserve. Changes in AMH may be influenced by systemic inflammatory status, treatment timing, sample size, model-related variability, or baseline lesion burden. Although baseline lesion area was numerically larger in the Fetuin-A group, the primary lesion-area analysis adjusted for baseline lesion area using ANCOVA; nevertheless, baseline disease burden remains relevant when interpreting AMH findings. Accordingly, this finding should be interpreted cautiously with respect to reproductive safety or ovarian toxicity.

These findings should be interpreted as evidence of treatment-associated molecular and histological modulation rather than proof of direct pathway mediation. After Benjamini–Hochberg false-discovery-rate adjustment across the secondary biological endpoint family, all 12 biochemical, qRT-PCR, and immunohistochemical comparisons remained statistically significant (Supplementary Table S1). Eleven of these comparisons also remained below 0.05 after Bonferroni correction; however, multiplicity adjustment reduces but does not eliminate false-positive and selective-reporting concerns, particularly given the small sample size. Although HMGB1, OPN, p62/SQSTM1, VEGF, SOD1, and caspase-3 provide insight into inflammatory, remodeling, oxidative, autophagy-associated, angiogenic, and apoptotic processes, broader pathway validation would be required to define the precise mechanisms underlying the observed effects.

Despite these methodological boundaries, the consistency across systemic cytokines, HMGB1 expression, and immunohistochemical markers supports the biological coherence of the findings. The data suggest that Fetuin-A may shift the endometriotic lesion environment away from a pro-inflammatory, pro-angiogenic state and toward a less inflammatory remodeling phenotype. Future studies should evaluate dose response, treatment duration, lesion size, fertility outcomes, and recurrence in rat models and should directly test whether HMGB1 blockade or restoration modifies the effects of Fetuin-A. Such experiments would help clarify whether HMGB1-associated signaling is a primary mediator of Fetuin-A activity in experimental endometriosis or part of a broader anti-inflammatory response.

## 5. Conclusions

In conclusion, intraperitoneal Fetuin-A administration attenuated experimental endometriosis, with a significant baseline-adjusted reduction in the primary lesion-area endpoint, suppressing systemic inflammatory cytokines and downregulating *HMGB1* mRNA expression within endometriotic implants. These effects were accompanied by decreased VEGF and OPN immunohistochemical staining and increased Fetuin-A, p62/SQSTM1, caspase-3, and SOD1 immunoreactivity, suggesting coordinated modulation of inflammatory, angiogenic, oxidative stress, autophagy-associated, apoptotic, and tissue-remodeling marker responses. Although the AMH finding, the divergent *OPN* mRNA/protein pattern, and the absence of direct fibrosis-marker assessment require cautious interpretation, the overall findings support Fetuin-A as a biologically relevant modulator of inflammatory and lesion-remodeling responses associated with *HMGB1* transcript-level changes in endometriosis, but they do not establish direct HMGB1 pathway inhibition or definitive anti-fibrotic activity. Further studies are warranted to define the underlying mechanisms, clarify reproductive safety, evaluate dose and duration effects, and determine whether targeting Fetuin-A–HMGB1-related pathways may have translational potential for endometriosis treatment.

## Figures and Tables

**Figure 1:**
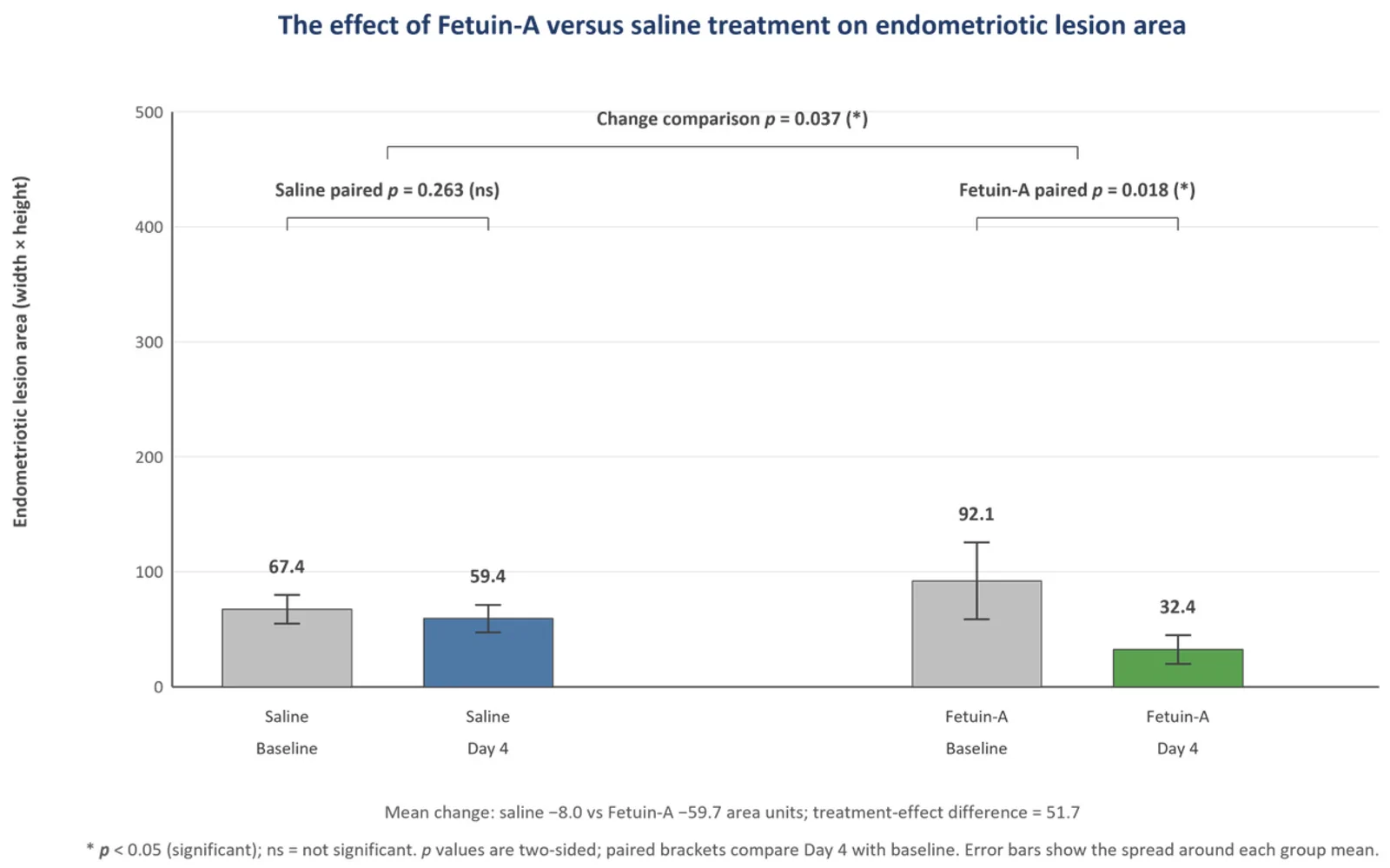
Primary lesion-area endpoint analysis of Fetuin-A versus saline treatment on endometriotic lesion area after four days. Endometriotic lesion area was calculated as width × height at baseline and after treatment. Bars represent mean ± SEM.

**Figure 2:**
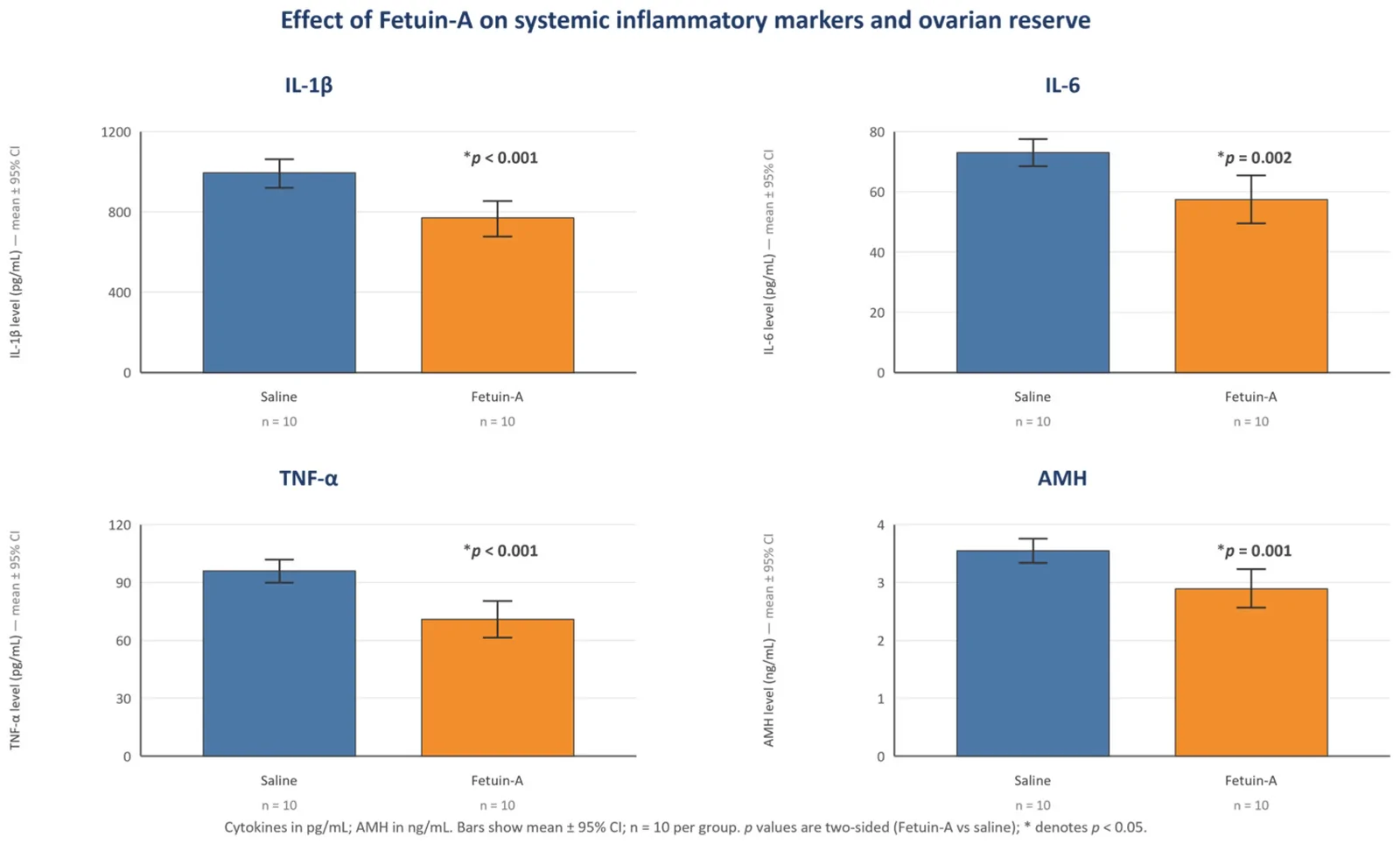
Effect of Fetuin-A treatment on systemic inflammatory markers and AMH in a rat model of endometriosis. Bars represent group mean serum concentrations of IL-1β, IL-6, TNF-α (pg/mL), and AMH (ng/mL) in the saline and Fetuin-A groups (n = 10 per group). Error bars indicate 95% confidence intervals.

**Figure 3:**
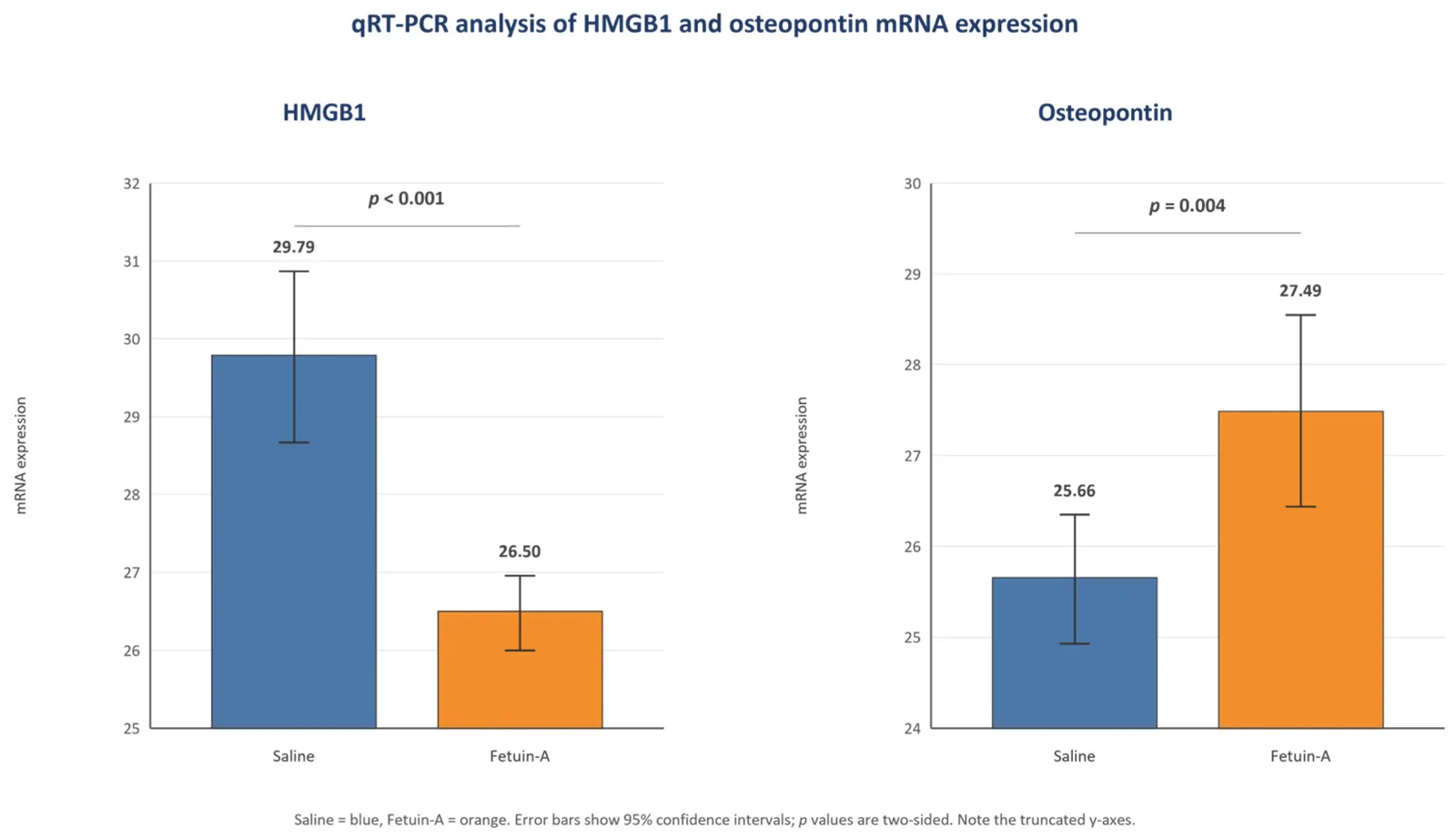
qRT-PCR analysis of *HMGB1* and osteopontin (*OPN*) mRNA expression in endometriotic tissue. Bars represent group mean expression levels in the saline and Fetuin-A groups, and error bars indicate 95% confidence intervals.

**Figure 4:**
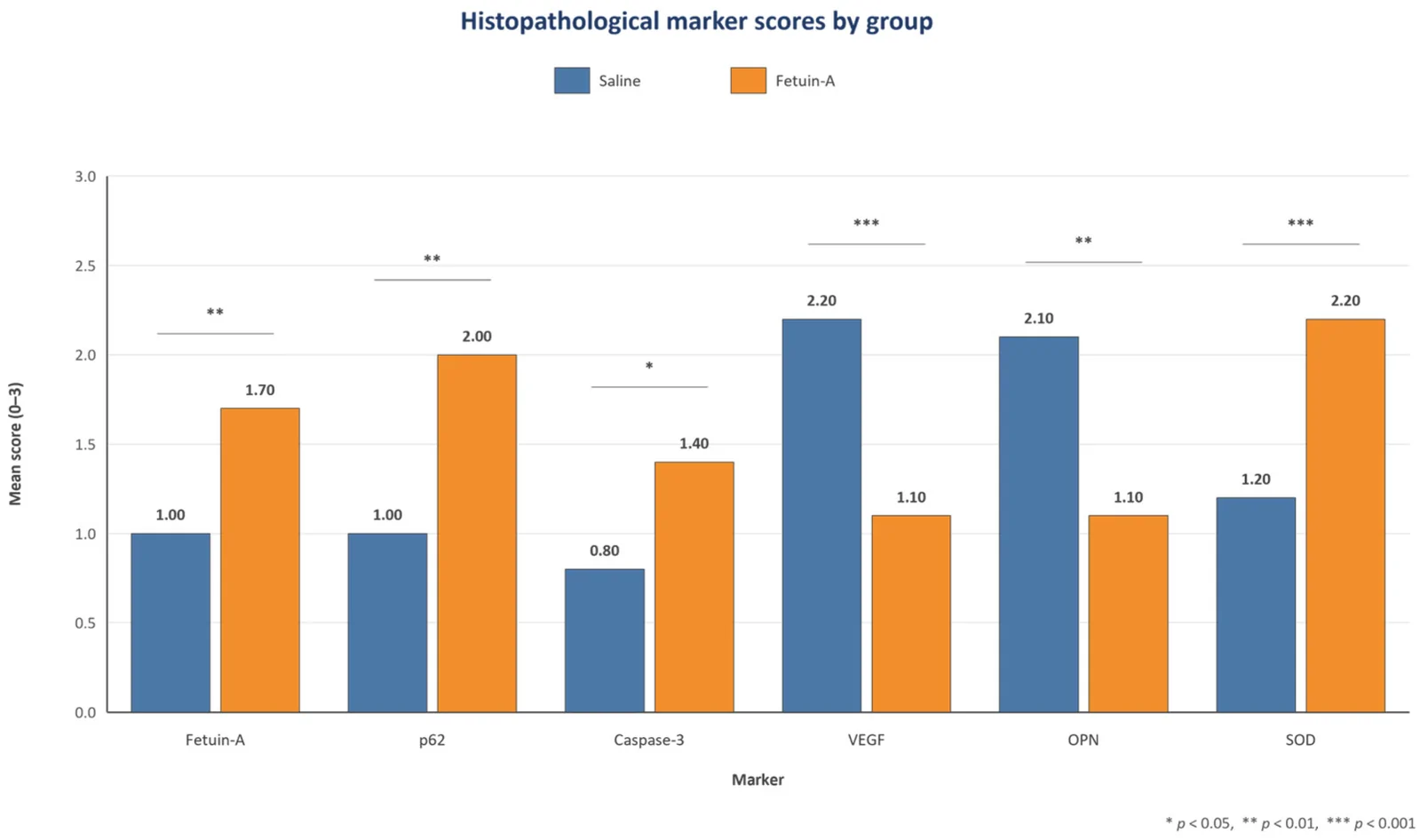
Semi-quantitative immunohistochemical marker scores in saline-treated and Fetuin-A-treated groups. Bars represent group mean marker scores; higher scores indicate stronger immunohistochemical staining intensity according to the applied scoring system.

**Table 1:** Primer sequences used for quantitative real-time PCR analysis.

Gene Symbol	Primer Direction	Sequence (5′ → 3′)	Product Size
*HMGB1*	Forward	GATGGGCAAAGGAGATCCTAAG	124 bp
	Reverse	TCACTTTTTGTCTCCCCTTGGC	
*OPN*	Forward	GAGGAGAAGGCGCATTACAG	153 bp
	Reverse	GGACGATTGGAGTGAAAGTG	
β-actin (*ACTB*)	Forward	AGCCATGTACGTAGCCATCC	115 bp
	Reverse	ACCCTCATAGATGGGCACAG	

## Data Availability

The raw data supporting the conclusions of this article will be made available by the authors on request.

## Supplementary Materials

The following supporting information can be downloaded at https://www.mdpi.com/article/10.3390/biomedicines14102149/s1: Table S1: Multiplicity-adjusted secondary biological endpoint comparisons, including raw *p*-values, Benjamini–Hochberg false-discovery-rate-adjusted *p*-values, and Bonferroni-adjusted *p*-values for the 12 biochemical, qRT-PCR, and immunohistochemical comparisons treated as one secondary biological endpoint family; Figure S1: Representative immunohistochemical staining of Fetuin-A expression in endometriotic implants; Figure S2: Representative immunohistochemical staining of osteopontin (OPN) expression in endometriotic implants; Figure S3: Representative immunohistochemical staining of p62 expression in endometriotic implants from the saline group; Figure S4: Representative immunohistochemical staining of p62 expression in endometriotic implants from the Fetuin-A group; Figure S5: Representative immunohistochemical staining of caspase-3 expression in endometriotic implants; Figure S6: Representative immunohistochemical staining of SOD1 expression in endometriotic implants; Figure S7: Representative immunohistochemical staining of VEGF expression in endometriotic implants.

## Abbreviations

AMH: anti-Müllerian hormone; ARRIVE: Animal Research: Reporting of In Vivo Experiments; BAUN-DEHAM: Balıkesir University Experimental Animals Production, Care, Application and Research Center; ELISA: enzyme-linked immunosorbent assay; HMGB1: high mobility group box 1; IL: interleukin; OPN: osteopontin; qRT-PCR: quantitative real-time polymerase chain reaction; RAGE: receptor for advanced glycation end products; SOD1: superoxide dismutase 1; TNF-α: tumor necrosis factor-alpha; VEGF: vascular endothelial growth factor.
