# Supplementary material for: Intraperitoneal Fetuin-A Modulates HMGB1-Associated Inflammatory Remodeling in Experimental Endometriosis
Source: Biomedicines. 2026 Sep 23;14(10):2149. doi: 10.3390/biomedicines14102149 (PMC13701466; doi:10.3390/biomedicines14102149)
Supplement: Supplementary file 1 [file biomedicines-14-02149-s001.zip › biomedicines-4564160-supplementary.pdf]

Supplementary Materials:

Supplementary Table S1. Multiplicity-adjusted secondary biological endpoint comparisons. Raw p values, Benjamini–Hochberg false-discovery-rate adjusted p values, and Bonferroni-adjusted p values are shown for the 12 biochemical, qRT-PCR, and immunohistochemical comparisons treated as one secondary biological endpoint family. The baseline-adjusted lesion-area ANCOVA was defined as the primary endpoint analysis and is reported in the main Results section.

Endpoint	Raw p value	BH-FDR adjusted p	Bonferroni adjusted p
Serum cytokines and ovarian reserve			
**IL-1β serum**	0.0003	0.0015	0.0030
**IL-6 serum**	0.0016	0.0023	0.0187
**TNF-α serum**	0.0000	0.0005	0.0005
**AMH serum**	0.0013	0.0023	0.0161
qRT-PCR			
**HMGB1 mRNA**	0.0010	0.0023	0.0120
**OPN mRNA**	0.0040	0.0044	0.0480
IHC marker scores			
**Fetuin-A**	0.0013	0.0023	0.0159
**p62/SQSTM1**	0.0035	0.0042	0.0424
**Caspase-3**	0.0110	0.0110	0.1323
**VEGF**	0.0007	0.0023	0.0084
**OPN**	0.0035	0.0042	0.0415
**SOD1**	0.0008	0.0023	0.0092

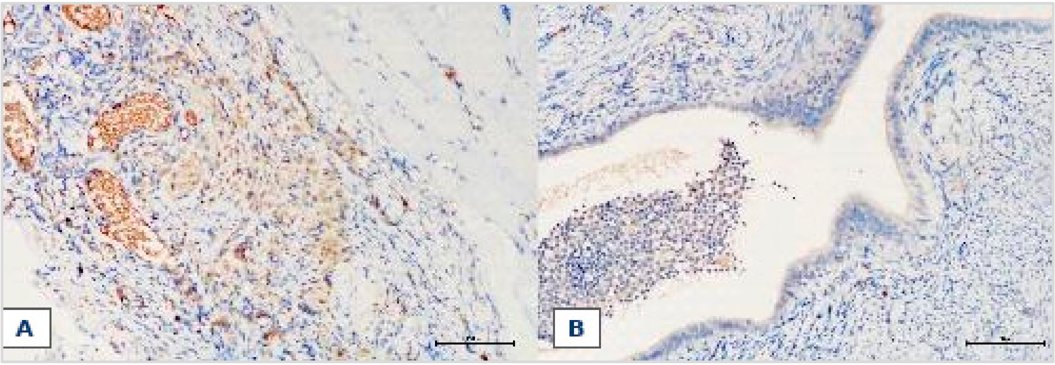

Supplementary Figure S1. Representative immunohistochemical staining of Fetuin-A expression in endometriotic implants. (A) Moderate cytoplasmic Fetuin-A expression (2+) in stromal cells of endometriotic lesions from the Fetuin-A group (×200). (B) Weak cytoplasmic Fetuin-A expression (1+) in the glandular epithelium of endometriotic lesions from the saline group (×200).

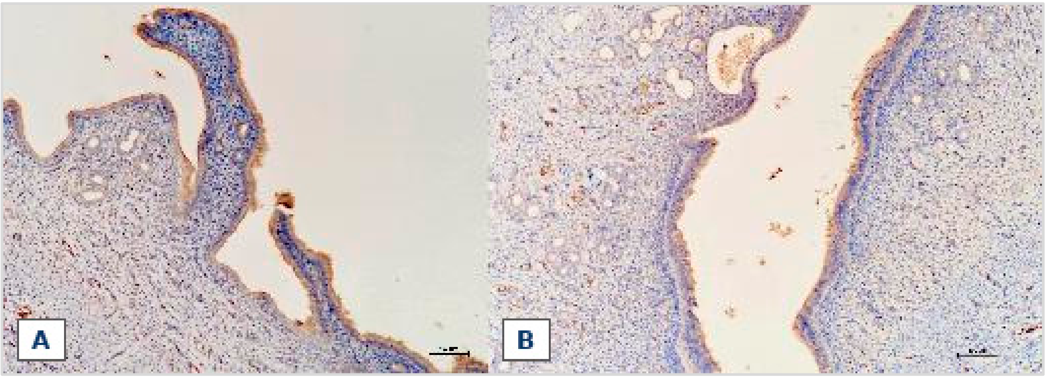

Supplementary Figure S2. Representative immunohistochemical staining of osteopontin (OPN) expression in endometriotic implants. (A) Strong cytoplasmic OPN expression (3+) in the glandular

epithelium of endometriotic lesions from the saline group (×100). (B) Moderate cytoplasmic OPN expression (2+) in the glandular epithelium of endometriotic lesions from the Fetuin-A group (×100).

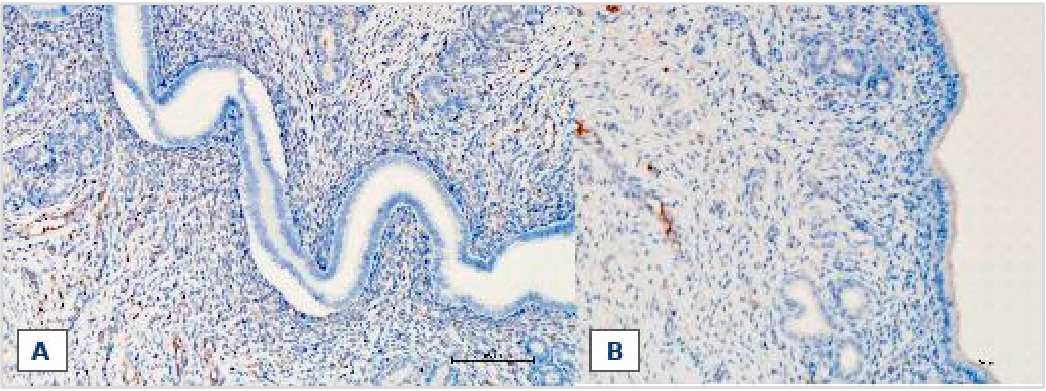

Supplementary Figure S3. Representative immunohistochemical staining of p62 expression in endometriotic implants from the saline group. (A) Absence of cytoplasmic p62 expression in the glandular epithelium (×200). (B) Weak cytoplasmic p62 immunoreactivity (1+) in the glandular epithelium (×200).

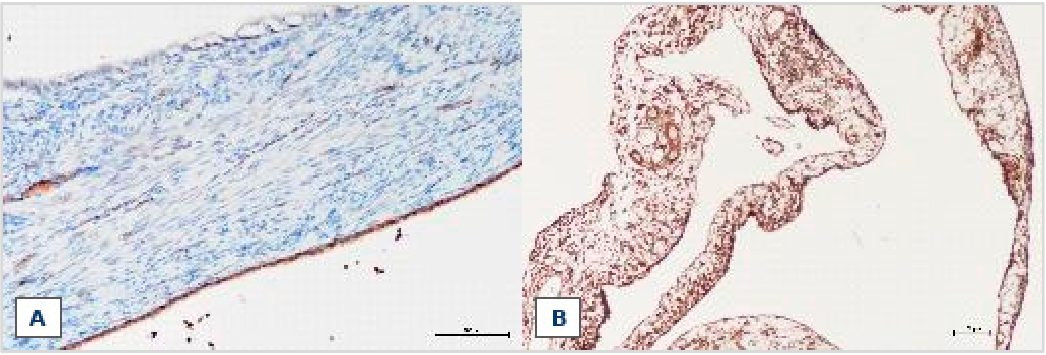

Supplementary Figure S4. Representative immunohistochemical staining of p62 expression in endometriotic implants from the Fetuin-A group. (A) Moderate cytoplasmic p62 expression (2+) in the glandular epithelium (×200). (B) Strong cytoplasmic p62 expression (3+) in the glandular epithelium (×200).

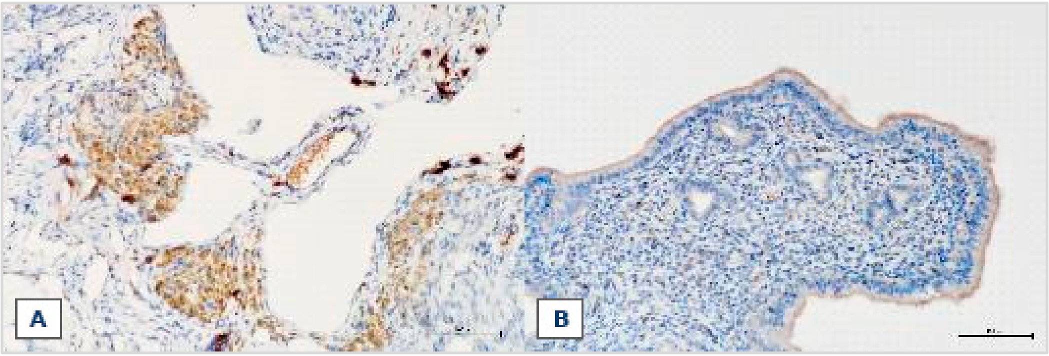

Supplementary Figure S5. Representative immunohistochemical staining of caspase-3 expression in endometriotic implants. (A) Moderate cytoplasmic caspase-3 expression (2+) in stromal cells of the Fetuin-A group. (B) Weak caspase-3 immunoreactivity (1+) in the glandular epithelium of the saline group. Original magnification, ×200.

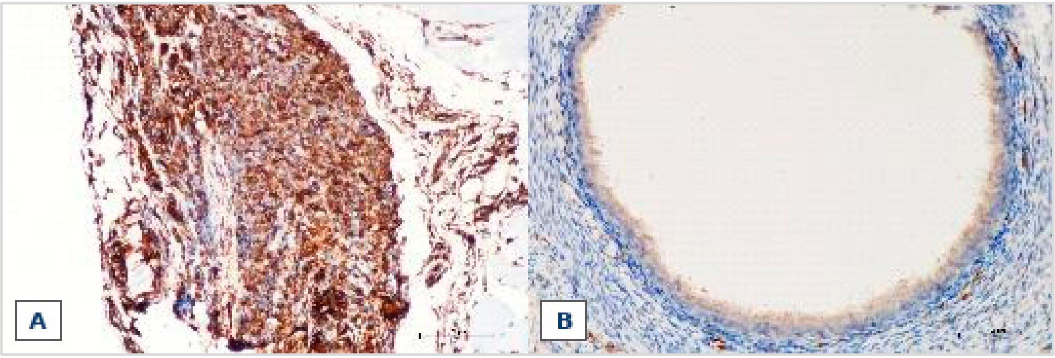

Supplementary Figure S6. Representative immunohistochemical staining of SOD1 expression in endometriotic implants. (A) Strong cytoplasmic SOD1 expression (3+) in stromal cells and moderate expression (2+) in the glandular epithelium of the Fetuin-A group. (B) Weak SOD1 immunoreactivity (1+) in the saline group. Original magnification, ×200.

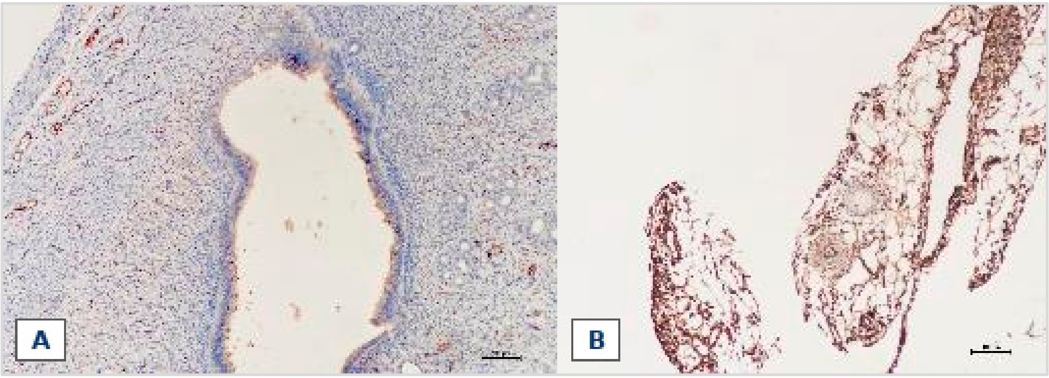

Supplementary Figure S7. Representative immunohistochemical staining of VEGF expression in endometriotic implants. (A) Reduced VEGF immunoreactivity in the Fetuin-A group. (B) Moderate to strong cytoplasmic VEGF expression (2–3+) in the glandular epithelium of the saline group. Original magnification, ×100.
